# Supplementary material for: Mayaro virus pathogenesis and immunity in rhesus macaques
Source: PLoS Negl Trop Dis. 2023 Nov 20;17(11):e0011742. doi: 10.1371/journal.pntd.0011742 (PMC10695392; doi:10.1371/journal.pntd.0011742)
Supplement: S1 Table — Presence and relative intensity (+ to +++) of lymphocytic inflammation within tissues were assessed using the following scale:—, absence of pathology within sections; +, one small aggregate of perivascular lymphocytes; ++, multiple blood vessels within one or two areas of the tissue with small to moderate numbers of perivascular lymphocytes; +++, perivascular lymphocytes affecting a majority of blood vessels in small to moderate numbers with or without infiltration of the surrounding tissue. *, Chronic hepatic degeneration and regeneration; **, Rare attenuated cortical tubules, scant cellular or proteinaceous casts with few associated lymphocytes; and ***, diffuse chronic mild lymphocytic and neutrophilic urethritis. (DOCX) [file pntd.0011742.s001.docx]

**S1 Table. Presence or absence of perivascular lymphocytic inflammation in endocrine, respiratory, alimentary, hepatobiliary and pancreatic, and genitourinary tissues in MAYV-infected rhesus macaques at 10 dpi.** Tissues are scored for presence of lymphocytic inflammation by relative intensity (+ to +++) or absence (-) of pathology within sections.

| **Tissue**​ | **Animal 28472**​ | **Animal 30504**​ | **Animal 36647**​ |
| --- | --- | --- | --- |
| **Endocrine**​ | | | |
| Thyroid gland​ | -​ | -​ | -​ |
| **Respiratory**​ ​ | | | |
| Lungs​ | -​ | -​ | -​ |
| **Alimentary**​​ | | | |
| Parotid salivary gland​ | -​ | -​ | -​ |
| Submandibular salivary gland​ | -​ | -​ | -​ |
| Duodenum​ | -​ | -​ | -​ |
| Jejunum​ | -​ | -​ | -​ |
| Ileum​ | -​ | -​ | -​ |
| Cecum​ | -​ | -​ | -​ |
| Colon​ | -​ | -​ | -​ |
| **Hepatobiliary and pancreatic**​ ​ | | | |
| Liver​ | -​ | ++​ *​ | ++​ |
| Gall bladder​ | -​ | -​ | -​ |
| Pancreas​ | -​ | -​ | -​ |
| **Genitourinary**​ ​ | | | |
| Kidneys​ | + ** | -​ | -​ |
| Urinary bladder​ | -​ | -​ | -​ |
| Urethra​ | -​ | -​ | +++​ ***​ |
| Prostate​ | -​ | +​ | -​ |
| Seminal vesicles​ | -​ | -​ | -​ |
| Epididymis​ | -​ | -​ | +​ |
| Testes​ | -​ | -​ | -​ |

+, one small aggregate of perivascular lymphocytes; ++, multiple blood vessels within one or two areas of the tissue with small to moderate numbers of perivascular lymphocytes; +++, perivascular lymphocytes affecting a majority of blood vessels in small to moderate numbers with or without infiltration of the surrounding tissue.

*, Chronic hepatic degeneration and regeneration; **, Rare attenuated cortical tubules, scant cellular or proteinaceous casts with few associated lymphocytes; ***, diffuse chronic mild lymphocytic and neutrophilic urethritis.
