# Supplementary material for: Mayaro virus pathogenesis and immunity in rhesus macaques
Source: PLoS Negl Trop Dis. 2023 Nov 20;17(11):e0011742. doi: 10.1371/journal.pntd.0011742 (PMC10695392; doi:10.1371/journal.pntd.0011742)
Supplement: S2 Table — Table summarizes pathologic diagnoses in given lymphoid tissues. Absence of observed pathology within the tissue is denoted (-). (DOCX) [file pntd.0011742.s002.docx]

**S2 Table. Hematopoietic pathology in MAYV-infected rhesus macaques at 10 dpi.** Table summarizes pathologic diagnoses in given lymphoid tissues. Absence of observed pathology within the tissue is denoted (-).

| **Tissue**​ | **Animal 28472**​ | **Animal 30504**​ | **Animal 36647**​ |
| --- | --- | --- | --- |
| Bone marrow​ | -​ | -​ | -​ |
| Thymus​ | -​ | -​ | -​ |
| Spleen​ | Perifollicular sinusoid congestion, sinusoidal reticuloendothelial hyperplasia, increased neutrophils within the red pulp​ | Perifollicular sinusoid congestion, sinusoidal reticuloendothelial hyperplasia, increased neutrophils within the red pulp​ | Perifollicular sinusoid congestion, sinusoidal reticuloendothelial hyperplasia, increased neutrophils within the red pulp​ |
| Cervical lymph node​ | Sinus histiocytosis with hemosiderophages, minimal​ | -​ | N​ |
| Submandibular lymph node​ | -​ | -​ | Sinus histiocytosis, mild​ |
| Axillary lymph node​ | Sinus histiocytosis and plasmacytosis, mild, with hemosiderophages​ | Follicular hyperplasia, minimal, axillary lymph node ​  Sinus histiocytosis, with hemosiderophages, axillary lymph node ​ | Lymphofollicular hyperplasia, moderate, with moderate sinus histiocytosis and plasmacytosis with hemosiderophages​ |
| Tracheobronchial lymph node​ | -​ | Follicular hyperplasia, mild​ | Follicular hyperplasia, multifocal, mild​ |
| Retroperitoneal lymph node​ | -​ | -​ | -​ |
| Mesenteric lymph node​ | Sinus histiocytosis and plasmacytosis, mild, with hemosiderophages​ | -​ | Sinus histiocytosis, moderate ​  Follicular hyperplasia, mild​ |
| Iliosacral lymph node​ | Sinus histiocytosis, mild, with hemosiderophages​ | -​ | Sinus histiocytosis and plasmacytosis with hemosiderophages, mild ​ |
| Inguinal lymph node​ | -​ | Sinus histiocytosis, mild​ | Sinus histiocytosis, mild​ |
