# Supplementary material for: Mayaro virus pathogenesis and immunity in rhesus macaques
Source: PLoS Negl Trop Dis. 2023 Nov 20;17(11):e0011742. doi: 10.1371/journal.pntd.0011742 (PMC10695392; doi:10.1371/journal.pntd.0011742)
Supplement: Attachment — Submitted filename: PNTD-D-23-00708 MAYV NHP review.docx [file pntd.0011742.s015.docx]

The manuscript “Mayaro virus pathogenesis and immunity in rhesus macaques” by Weber *et al*. describes infection of, virus tropism, histopathology, and immunologic changes in 3 male rhesus macaques of different ages that were inoculated with a strain of MAYV isolated in Argentina. The virus strain was selected from a panel of strains that were first characterized in preliminary studies using 2 mouse models. Aside from a study in 1967 demonstrating susceptibility, non-human primate models of Mayaro virus do not exist; as such, this paper represents an important first step towards establishing one. Having a NHP model of MAYV is needed for evaluating countermeasures like vaccines and understanding infection dynamics of potential reservoir species like rhesus macaques. The introduction is comprehensive, the breadth of analyses performed is exhaustive, and the conclusions are generally supported by the data (see a few exceptions below). Figure 10 that shows the antibody data is not possible to evaluate given that panels from 2 figures are superimposed on top of each other in both the PDF and eps files provided. In addition, there are several major concerns as well as some minor concerns that could be considered to increase readability, transparency, and scientific rigor.

Major Comments

The manuscript overstates the findings given that only 3 rhesus macaques were used in the study and all were male; this limitation is mentioned only in the last paragraph of the discussion. The abstract, methods, and results should all make this design absolutely clear and also hedge the findings based on this small sample size that does not include female animals. Similarly, only young female C57BL/6 mice were used in the virus strain selection studies; this should be made clear in the results instead of just the methods. The manuscript could also include a rationale justifying use of just one sex for both mice and NHP. The range of NHP ages selected should also be mentioned in more than just the methods and the rationale could be explained.

A rationale for inoculating virus at 5 locations on each NHP arm could help the reader understand this approach.

It is not clear if mock-inoculated mice were included as controls for weight loss. It also does not seem that the NHP studies used mock-inoculated controls; could the weight loss in animal 30504 stem from daily sedation and experimental procedures?

The passage histories of the virus stocks are not provided in the methods. It is not clear if MAYV BeAr has a more extensive vertebrate cell or suckling mouse brain passage history that could lead to higher murine infectivity and dissemination compared to the other strains tested, which would be artifactual. Although it makes sense that choosing the most murine infectious strain would be more likely to elicit clinical disease in NHP, if enhanced virulence of MAYV BeAr stems from its passage history, that makes it less representative of wild type MAYV.

The viremia rebound in 2 of the NHP on 10 dpi seems unexpected, at least based on CHIKV or other alphavirus NHP studies (although perhaps MAYV is not like CHIKV or the other viruses in this respect). This observation would be more strongly supported if validation data was provided, i.e. repeat testing of a replicate 10 dpi plasma sample. Could the fact that no infectious virus detected on 10 dpi in plasma despite 10^4-5^ genomes/ml MAYV RNA suggest these were false MAYV RNA positives? Alternately, could the authors comment on whether the genome:PFU ratio could be in 100,000:1? The discrepancy between no infectious virus and 10^4-5^ genomes/ml RNA could be clarified, especially by showing the LOD of the plaque assays. With respect to rigor and reproducibility in general, neither the methods, results, or figure legends indicate how many replicate measurements were performed for the MAYV RT-qPCR data presented throughout the paper.

Detection of MAYV RNA in many nervous system tissues seems notable. Did any of the NHP show signs of neurologic disease? Along those lines, the results does not mention whether any other signs of disease besides the rash in 1 animal were observed, including any markers or proxies for arthritic disease.

Data should be provided in an open access repository instead of ‘upon request’.

**Minor Comments**

‘Chikungunya’ is not capitalized per ICTV as the virus is not named after a place.

Suggest adding CDC, WHO, and ECDC citations in the introduction in the same format as other citations.

Line 70: Evidence supporting rodent and NHP vertebrate hosts could be referenced with a citation, e.g. https://doi.org/10.3201/eid0910.030161.

Lines 72-77: Reference 15 and other vector competence studies for MAYV show that the threshold for infection of *Aedes aegypti* feeding on artificial bloodmeals is very high. Most of these studies use >10^6^ PFU/ml in bloodmeals, making *Aedes* cycles less likely if MAYV viremias in people or NHP are not that high. An advance of this study could be highlighted with respect to understanding viremias; NHP can help define the viremia profile and peak.

Line 93-94: It is also possible that MAYV is often not on the top differential for clinicians, especially in DENV endemic areas? Is it know how widespread MAYV diagnosis is attempted in MAYV endemic areas?

Line 141: ‘We subcutaneously infected’. Use of the word ‘infected’ presumes that mice become infected, which is not a surety. Consider ‘inoculated’. Same for line 168 with NHP.

Line 149: “Viral RNA levels were significantly higher for MAYVBeAr505411 with infection in muscles (ranging 1-3 logs higher) and joints (ranging 2-5 logs higher) compared to the other strains (Fig 1B-1G).” and Line 153 “Viral RNA levels in MAYVCH and MAYVGuyane infected C57BL/6 mice were similar to each other and higher than MAYVTRVL, MAYVUruma, or UNAVMAC, but still lower relative to MAYVBeAr505411 (Fig 1A-1H).” With the statistics shown, MAYVBeAr does not appear to be significantly higher than MAYV Guyane in multiple of the muscles and joints tested.

Line 165: “To characterize MAYV pathogenesis in NHPs, we infected three male rhesus macaques (RM) ages 4, 10, and 13 years (Fig 2).” Figure 2A does not show Ns or NHP ages.

Figure 2 (and throughout) would be more informative if the animal labels were ages instead of primate center identifiers.

Figure 2D: Is there a reason the Y-axis scale goes down to 70% when no animal dipped below 90%? Could it be re-set to have the lowest measurement around 85%?

Line 182: RE: “None of the three animals experienced weight loss over the duration of the 10 day infection study (Fig 2D).” The criteria for making this statement and statistical analyses should be shown as it looks like animal 30504 lost 10% of day 0 starting weight and held at that weight for all 10 days?

Line 187: “Viral supernatants were titered by limiting dilution plaque assays to quantify infectious viral particles in cell supernatants.” The word ‘quantify’ is confusing in this legend as only qualitative data are shown in the Table.

Figure 2B seems to have an added floating symbol on top of the legend.

Line 436: Have murine MAYV models evaluated whether rash develops, especially if shaving is not common for mice? If not, it is probably a stretch to say that the macaque is ‘a uniquely significant model for investigating dermatologic presentations’, especially with only one of 3 NHP developing a rash.

Line 443: RE: “However, in CHIKV infections in humans, it has been established that pre-existing chronic conditions are associated with increased inflammation and worsened disease [103, 104].” The methods do not clarify whether the 3 NHP used in this study were healthy or had pre-existing conditions that could have augmented disease severity.
