# Supplementary material for: Mayaro virus pathogenesis and immunity in rhesus macaques
Source: PLoS Negl Trop Dis. 2023 Nov 20;17(11):e0011742. doi: 10.1371/journal.pntd.0011742 (PMC10695392; doi:10.1371/journal.pntd.0011742)
Supplement: Attachment — Submitted filename: MAYV_NHP_rebuttal_PLOS_NTD_v2.docx [file pntd.0011742.s016.docx]

**RE: Mayaro virus pathogenesis and immunity in rhesus macaques**

To the PLOS NTD editors and reviewers,

We would like to thank the reviewers for their comprehensive evaluation of our manuscript and we are now excited to share our response paired with a significantly revised manuscript describing Mayaro virus pathogenesis and immunity in rhesus macaques. We have made changes to the manuscript that reflect the reviewers’ comments. In addition, we have added a new transcriptomic data and analysis of longitudinal macaque PBMC samples in order to characterize the global effects following MAYV infection (new figures 9, S10). A rebuttal for specific questions and comments raised by reviewers is included below.

**Authors’ responses to reviewer comments**

Reviewer #1: I have a number of minor suggestions/editorial edits:

- Line 24: add (NHP) after non-human primates as this is the first time the abbreviation would be used.

This abbreviation has been added.

- Line 28: You can mention here the animal number of n=3.

Mention of three animals in the abstract has been added to the manuscript.

- Lines 72-73: references 12-15 look to be studies in the laboratory (instead of in the real world), so it would be good to check this and mention it accordingly.

We have clarified that these studies were in research settings in the text. Reviewer 2 also asked us to clarify that transmission potential by urban-dwelling species was in context of experimental infection.

- Line 158: I assume that instead of immunocompetent, this should be immunodeficient?

This correction has been made.

- Line 164. In the section on MAVY replication kinetics, it would be good to also mention if any animals displayed any overt signs of disease or discomfort (e.g. joint pain). As the authors do not mention it, I assume nothing was observed except for the rash mentioned later, but it would be good to state this in the manuscript. An option is to even start with a separate section with the experimental design and clinical monitoring that can even include the information currently in lines 178-183  (CBC, fever and weight).

Animals did not display signs of disease (other than fever n=1, rash n=1) or discomfort and we have attempted to better emphasize this in the manuscript.

- Line 180: "...anemia that coincided with peak viremia....". It looks that anemia was more a gradual decline rather than coinciding with peak viremia. This may be related to the frequent sedations and blood collections that may have been close to the limit for this animal?

It is possible that the anemia is associated with frequent sedation and blood collections.

- Table 1: as this table includes many joints, can the authors clarify, perhaps in the materials and methods, whether when joint tissues were collected, the focus was on mostly joint capsule, rather than bone or cartilage, to test for virus levels?

Joint collections included tissue from the synovium, articular cartilage, joint capsule, and surrounding connective tissues.

- Lines 192 to 203: As this is a detailed description of skin pathology, this would fit better later in the manuscript where the rest of the pathology is described.

We chose to include the description of skin pathology at the point in the manuscript because it details clinical signs of disease that were identified in our study.

- Line 253: the statement "...and variations were absent microscopically where present on sections stained with...". Do the authors mean "macroscopically"?

This has been corrected.

- Line 276: fix the typo in "innate"

This correction has been made.

- As listed earlier, Figure 10 that was in the pdf to review is distorted.

The figure format has been fixed.

- Line 448: As the authors bring up the liver, did they monitor the animals in this study with any serum chemistry panels? If they did, they can mention it in the results. Otherwise no need to test the samples as the likelihood to find something significant is small.

We did monitor the three animals by serum chemistry panels and have now included that data in the manuscript in new supplemental figure 3, although we did not find any remarkable changes following infection.

- Lines 487-488: I suggest to also add long term pathological changes as a benefit of doing a study with longer duration.

We have added this sentence here that also gets at another comment from reviewer 2: “Although we did not detect signs of arthritic or neurologic disease, we hypothesize that there is potential for disease development beyond 10 dpi in this model evidenced by our tissue tropism data; disease and long-term pathological changes should be explored in longer future studies”

- Line 540: As the results mentioned the inoculum was administered on both arms, it is good to also mention it here.

We agree and have added these details to other sections of the manuscript as well.

- Line 772 : I don't feel the term hematopoietic is the best one to describe the figure, as the data are lymphoid pathology. A similar comment for line 798, but as there bone marrow is included, the header can include both.

This has been modified in the figure titles.

- This comment is optional for the authors whether or not to do this, but it looks that the manuscript uses actual animal ID numbers. To avoid getting FOIA requests about these animals, the authors can consider changing the animal numbers.

We have removed primate identifiers from figures and the manuscript and termed animals NHP 1-3.

**Reviewer #2:**

1. The manuscript is too extensive and needs to be trimmed. For example, the Introduction section is too long and repetitive. Please revise this section and keep only the essential contextualization for this study. Also, the Result section could be more limited to findings in the study (i.e., only results). Comparison with other studies should be explored in the Discussion section. Please be concise; this will help better understand of study and improve the readership.

We have trimmed the intro to make it more concise without taking away from what we feel is important background. We have removed some wording from the results, although we feel some references are important for contextualization of the data.

2. Please clarify why was selected these MAYV strains. This represents the different genotypes described in the Introduction section. Or was this chose was arbitrary and random? Please explain the rationale.

We attempted to evaluate several viruses across the phylogeny for D and L genotype MAYV strains but did not have access to a genotype N strain. We evaluated all of the MAYV strains available to us.

3. The titles in the Result section should be redone to summarize the main findings presented in each section (please see also in minor comments). This can call more attention to readers.

Titles of the results sections have been redone to better focus on section conclusions.

4. Consider including in the Discussion section the correlation between CNS and heart infection reported in clinical outcomes and experimental chikungunya virus infection. Please see below some examples of references that can help to contextualize it:

Noval et al. MAVS signaling is required to prevent persistent chikungunya heart infection and chronic vascular tissue inflammation. Nat Commun. 2023 Aug 3;14(1):4668. doi: 10.1038/s41467-023-40047-w. PMID: 37537212; PMCID: PMC10400619.

These references have been added following this sentence in the intro “Neurological complications associated with more severe cases and myocarditis has been reported following CHIKV infection, thus cardiac involvement has been hypothesized for other arthritogenic alphavirus infections including MAYV [41-47].”

This sentence with these citations have also been added to the discussion “Our evidence of viral detection and lymphocyte aggregation near rare blood vessels of the heart and central nervous tissues is in agreement with CHIKV findings in the context of clinical outcomes and experimental infection in animal models [45, 46].”

de Lima STS et al. Fatal Outcome of Chikungunya Virus Infection in Brazil. Clin Infect Dis. 2021 Oct 5;73(7):e2436-e2443. doi: 10.1093/cid/ciaa1038. PMID: 32766829; PMCID: PMC8492446.

5. Immunopathologic could be better explored, including brain and heart infection findings with MAYV (No comment in this current version). Also, I suggest immunoassaying for MAYV in brain and heart tissue to understand the potential replication sites in this tissue since this seems common in severe chikungunya cases.

The authors agree that this is an area that should be explored in the future and we mentioned this in the discussion section of the manuscript reading: “Future investigation into potential colocalization of viral particles and inflammatory or degenerative foci, which was not within the scope of this current study, would improve identification of lesion relevance.” This was a pilot study to establish the model and we had limited resources to conduct these experiments.

6. Please revise the main conclusion for this study for the most relevant findings supported by data (Lines 488-490). In short, the study contributes to understanding the pathogenesis and immunity. However, this impact seems more limited regarding the MAYV transmission, vaccine, monoclonal and antiviral.

These conclusions have been emphasized at the end of the paper in the final paragraph and edited at lines 488-490.

7. Mayaro strain BeAr505411 is not “Buenos Aires 505411” because this strain was isolated in Pará State, Brazil, in 1991 instead of “Buenos Aires, Argentina.”  The meaning of “BeAr” is Belém city in Pará State, Brazil, and “Ar” is “Arbovirology section,” where the Evandro Chagas Insitute has performed this viral isolation. Please correct it.

Thank you for this, we have corrected this error in the manuscript.

10. Consider a summary scheme to explain the main findings. This could help the reader understand the contribution of this work.

We think this is a great suggestion but feel that the manuscript has a great number of figures as is and have chosen to exclude this.

Figure 1. Consider including the experimental design as included in Figure 2.

A schematic has been added to figure 1.

Figure 2. In B, there is an extra triangle; please remove it. Since there are no body weight changes, consider move D for supplementary.

Panel B of figure 2 has been fixed and we have chosen to retain the body weight data in the main figures.

Figure 3. This could be moved for supplementary. We left this figure in as it clearly demonstrates an important clinical finding.

Figure 4. Please consider simplifying this figure in the main figures and moving details as supplementary figures. We would like to keep the detail in this figure as it demonstrates efficient spread of the virus in the NHP.

Figure 5. This could be moved for supplementary.

We have chosen to leave figures 3-5 in the main figures but have moved what was previously figure 9 (B cell analysis) to the supplement.

Figure 10. Multiple plots overlap. Please revise it.

This has been fixed.

Table 1. Since the virus was titred by plaque assay, please consider redoing this table as a plot using the titration instead of only positive and negative.

We have added titer quantities to Table 1 and have chosen not to graph this data due to low positivity rates.

Table 2. Consider replacing the table with a heatmap format to visualize this data better.

We have added color shading to help better visualize pathology findings in the table.

Minors:

Line 58: Replace “Western hemisphere” with “Latin American and Caribbean”

This change has been made in the manuscript.

Line 61: Delete “Western”.

This change has been made in the manuscript.

Line 61: Replace “Chikungunya” with “chikungunya”.

This change has been made in the manuscript.

Line 63: Consider replacing “WHO reference” with “Suhrbier A. et al. Nat Rev Rheumatol. 2019 Oct;15(10):597-611. doi: 10.1038/s41584-019-0276-9. Epub 2019 Sep 3. PMID: 31481759.”

Line 64: Consider replacing “ECDC reference” with “de Souza WM et al. Lancet Microbe. 2023 May;4(5):e319-e329. doi: 10.1016/S2666-5247(23)00033-2. Epub 2023 Apr 6. PMID: 37031687; PMCID: PMC10281060.

We have added these references.

Lines 66-67: Some studies evaluated the cross-reactions between both viruses, such as:

-	Webb et al. Sci Rep. 2019 Dec 31;9(1):20399. doi: 10.1038/s41598-019-56551-3. PMID: 31892710; PMCID: PMC6938517.

-	Fumagalli et al. J Virol. 2021 Nov 9;95(23):e0112221. doi: 10.1128/JVI.01122-21. Epub 2021 Sep 22. PMID: 34549980; PMCID: PMC8577356.

-	Martins et al. Neutralizing Antibodies from Convalescent Chikungunya Virus Patients Can Cross-Neutralize Mayaro and Una Viruses. Am J Trop Med Hyg. 2019 Jun;100(6):1541-1544. doi: 10.4269/ajtmh.18-0756. PMID: 31017081; PMCID: PMC6553899.

We have added these references.

Lines 70-72: Most findings related to Aedes mosquitoes being able to transmit MAYV are from experimental infection; please clarify that it is linked to experimental studies. For example:

Cereghino et al. PLoS Pathog. 2023 Apr 5;19(4):e1010491. doi: 10.1371/journal.ppat.1010491. PMID: 37018377; PMCID: PMC10109513.

Pereira et al. PLoS Negl Trop Dis. 2020 Apr 14;14(4):e0007518. doi: 10.1371/journal.pntd.0007518. PMID: 32287269; PMCID: PMC7182273.

We have added these references are rephrased this sentence. Reviewer 1 also asked us to clarify that transmission by other species was in context of experimental infection.

The manuscript overstates the findings given that only 3 rhesus macaques were used in the study and all were male; this limitation is mentioned only in the last paragraph of the discussion. The abstract, methods, and results should all make this design absolutely clear and also hedge the findings based on this small sample size that does not include female animals. Similarly, only young female C57BL/6 mice were used in the virus strain selection studies; this should be made clear in the results instead of just the methods. The manuscript could also include a rationale justifying use of just one sex for both mice and NHP. The range of NHP ages selected should also be mentioned in more than just the methods and the rationale could be explained.

We have clarified the animal selection in the text.

A rationale for inoculating virus at 5 locations on each NHP arm could help the reader understand this approach.

We inoculated at 5 locations on each NHP arm in an attempt to mimic infection through infected mosquito bites and have added these details to the results and methods of the manuscript. We have successfully used this for other arboviruses including CHIKV and ZIKV.

It is not clear if mock-inoculated mice were included as controls for weight loss. It also does not seem that the NHP studies used mock-inoculated controls; could the weight loss in animal 30504 stem from daily sedation and experimental procedures?

Mock inoculated mice were not included as controls for weight loss and neither were NHPs. The 7% weight loss observed for NHP 3 between the biopsy day and infection day could likely be due to sedation and experimental procedures.

The passage histories of the virus stocks are not provided in the methods. It is not clear if MAYV BeAr has a more extensive vertebrate cell or suckling mouse brain passage history that could lead to higher murine infectivity and dissemination compared to the other strains tested, which would be artifactual. Although it makes sense that choosing the most murine infectious strain would be more likely to elicit clinical disease in NHP, if enhanced virulence of MAYV BeAr stems from its passage history, that makes it less representative of wild type MAYV.

The authors agree that passage history could definitely impact virulence in mice and appreciate this comment. Upon receiving the viral stocks from BEI, viruses were passaged only once or twice in the lab before use in animal experiments or lab assays and we have included these details in the methods. On the BEI website, we found that MAYV_BeAr505411_ and MAYV_Uruma_ strains were each passaged once and the TRVL strain was passaged 3 times, all for removal of mycoplasma. For the MAYV_Guyane_ no passages are detailed in the BEI product resource. MAYV_CH_ was generated from an infectious clone so our lab stock is very low passage- p2. Indeed, it is possible that the reduced virulence we observed in mice for the MAYV_TRVL_ strain could be due to more extensive passage history but the other strains we evaluated were lower passage. We have added a comment in the manuscript that passage history could impact murine pathogenesis.

The viremia rebound in 2 of the NHP on 10 dpi seems unexpected, at least based on CHIKV or other alphavirus NHP studies (although perhaps MAYV is not like CHIKV or the other viruses in this respect). This observation would be more strongly supported if validation data was provided, i.e. repeat testing of a replicate 10 dpi plasma sample. Could the fact that no infectious virus detected on 10 dpi in plasma despite 10^4-5^ genomes/ml MAYV RNA suggest these were false MAYV RNA positives? Alternately, could the authors comment on whether the genome:PFU ratio could be in 100,000:1? The discrepancy between no infectious virus and 10^4-5^ genomes/ml RNA could be clarified, especially by showing the LOD of the plaque assays. With respect to rigor and reproducibility in general, neither the methods, results, or figure legends indicate how many replicate measurements were performed for the MAYV RT-qPCR data presented throughout the paper.

To ensure that our plasma vRNA data was reproducible, we have repeated the qRT-PCR for detection of MAYV vRNA in NHP plasma samples with two additional independent experiments and were able to validate our original findings with the exception of the 10 dpi positive samples. We have repeated the assay in three independent new experiments and have included what we feel is our most accurate viremia curve in figure 2 and below. All vRNA detection throughout the paper was completed in triplicate reactions for each experiment, and we have added these details to the manuscript.

We hypothesize that the infectious virus to vRNA ratio would be more consistent earlier on post-infection for the tissues and feel that our infectious virus detection data in plasma is rather consistent with the vRNA data. For the tissue infectious virus detection, due to sampling bias and timeframe after peak viremia, it is difficult to consistently detect infectious virus. The authors are open to suggestions of alternative approaches for isolation of infectious virus. We also attempted to titer the tissues collected in PBS on vero cells immediately but were unsuccessful in that approach, which is why we infected C6/36 cells with the tissue supernatants. We have added quantitative data to Table 1 as well as the LOD for the assay, which we previously excluded because these are viral titers of cellular supernatant after viral outgrowth.

Detection of MAYV RNA in many nervous system tissues seems notable. Did any of the NHP show signs of neurologic disease? Along those lines, the results do not mention whether any other signs of disease besides the rash in 1 animal were observed, including any markers or proxies for arthritic disease.

None of the NHPs showed any signs of neurologic or arthritic disease and we did not identify specific proxies for arthritis. Clarification that no additional signs of disease were observed in the macaques has been added to the manuscript. We did not expect to observe the development of arthritis within this study timeframe but we hope to further explore our samples for early signatures of arthritis in the future, such as in our transcriptomics dataset.

Data should be provided in an open access repository instead of ‘upon request’.

**Minor Comments**

‘Chikungunya’ is not capitalized per ICTV as the virus is not named after a place.

This has been corrected in the manuscript.

Suggest adding CDC, WHO, and ECDC citations in the introduction in the same format as other citations.

We have replaced these citations with alternative citations.

Line 70: Evidence supporting rodent and NHP vertebrate hosts could be referenced with a citation, e.g. <https://doi.org/10.3201/eid0910.030161>.

This reference has been added.

Lines 72-77: Reference 15 and other vector competence studies for MAYV show that the threshold for infection of *Aedes aegypti* feeding on artificial bloodmeals is very high. Most of these studies use >10^6^ PFU/ml in bloodmeals, making *Aedes* cycles less likely if MAYV viremias in people or NHP are not that high. An advance of this study could be highlighted with respect to understanding viremias; NHP can help define the viremia profile and peak.

The authors agree with this conclusion and have added a comment about this to the discussion.

Line 93-94: It is also possible that MAYV is often not on the top differential for clinicians, especially in DENV endemic areas? Is it know how widespread MAYV diagnosis is attempted in MAYV endemic areas?

Line 141: ‘We subcutaneously infected’. Use of the word ‘infected’ presumes that mice become infected, which is not a surety. Consider ‘inoculated’. Same for line 168 with NHP.

We have added “inoculated” or “challenged” at several locations in the manuscript.

Line 149: “Viral RNA levels were significantly higher for MAYVBeAr505411 with infection in muscles (ranging 1-3 logs higher) and joints (ranging 2-5 logs higher) compared to the other strains (Fig 1B-1G).” and Line 153 “Viral RNA levels in MAYVCH and MAYVGuyane infected C57BL/6 mice were similar to each other and higher than MAYVTRVL, MAYVUruma, or UNAVMAC, but still lower relative to MAYVBeAr505411 (Fig 1A-1H).” With the statistics shown, MAYVBeAr does not appear to be significantly higher than MAYV Guyane in multiple of the muscles and joints tested.

The authors agree that viral load was not higher in *all* cases for MAYV-BeAr infected mice compared to Guyane but do believe that this was the trend in many tissues and have softened the language around this in the manuscript to read “generally trended” and “for many tissues”.

Line 165: “To characterize MAYV pathogenesis in NHPs, we infected three male rhesus macaques (RM) ages 4, 10, and 13 years (Fig 2).” Figure 2A does not show Ns or NHP ages.

These details have been added to Figure 2A.

Figure 2 (and throughout) would be more informative if the animal labels were ages instead of primate center identifiers.

We have removed primate identifiers from figures and the manuscript and termed animals NHP 1-3.

Figure 2D: Is there a reason the Y-axis scale goes down to 70% when no animal dipped below 90%? Could it be re-set to have the lowest measurement around 85%?

We have adjusted the scale of the body weight change graph.

Line 182: RE: “None of the three animals experienced weight loss over the duration of the 10 day infection study (Fig 2D).” The criteria for making this statement and statistical analyses should be shown as it looks like animal 30504 lost 10% of day 0 starting weight and held at that weight for all 10 days?

The authors understand that ideally statistical analyses would be shown to support this conclusion, but the only way we could statistically analyze this data is if we grouped the three animals to get three data points to compare at each time point. We believe the reviewers are asking if we can show statistics for changes between timepoints for each animal, but this is not possible given that animals were not weighed three times, and thus there is only one data point for each animal at each timepoint. We have added to the text that NHP 3 did experience 7% loss of body weight between the biopsy period and infection day, but body weight held steady over the ten-day infection period. The authors are open to alternative analyses upon reviewer suggestion.

Line 187: “Viral supernatants were titered by limiting dilution plaque assays to quantify infectious viral particles in cell supernatants.” The word ‘quantify’ is confusing in this legend as only qualitative data are shown in the Table.

We have added quantitative data to the table.

Figure 2B seems to have an added floating symbol on top of the legend.

This has been removed.

Line 436: Have murine MAYV models evaluated whether rash develops, especially if shaving is not common for mice? If not, it is probably a stretch to say that the macaque is ‘a uniquely significant model for investigating dermatologic presentations’, especially with only one of 3 NHP developing a rash.

We understand why this statement could be considered a stretch and have reworded this sentence to read: “Maculopapular rash has not been reported in mice although it is possible it is missed due to lack of shaving, and biopsies of human specimens are not widely conducted, potentially making the macaque a uniquely significant model for investigating dermatologic presentations of MAYV.”

Line 443: RE: “However, in CHIKV infections in humans, it has been established that pre-existing chronic conditions are associated with increased inflammation and worsened disease [103, 104].” The methods do not clarify whether the 3 NHP used in this study were healthy or had pre-existing conditions that could have augmented disease severity.

The sole clinically apparent pre-existing condition present at the beginning of the study was dental disease in all three animals. While there were no relevant clinically apparent conditions prior to endpoint, age-related and incidental chronic lesions found at necropsy are described in the Results section on lines 272-276 and lines 482-483 as well as supplemental table 1.
